# Robot-Assisted Partial Splenectomy for Splenic Epidermoid Cyst

**DOI:** 10.1155/2020/6245909

**Published:** 2020-09-07

**Authors:** Mubarak Ali kirih, Xiao Liang, Yangyan Xie, Jingwei Cai, Junhao Zheng, Feng Xu, Shilin He, Liye Tao, Faisa Ali Abdi

**Affiliations:** Department of General Surgery, Sir Run Run Shaw Hospital, Zhejiang University School of Medicine, Hangzhou, Zhejiang Province, China

## Abstract

The splenic cyst is a rare disease with unknown etiology. The inner wall of the cyst has lining epithelium. The cyst can be unilocular or multilocular. According to pathology, it can be divided into four types: epidermoid cyst, dermoid cyst, cystic lymphangioma, and cystic hemangioma. Ultrasound examination is often the first choice for splenic cysts because of its nonradiation, low cost, and convenient examination. The images are mostly cystic masses with clear borders and dark areas without echoes, after the detection of splenic space-occupying lesions by ultrasonography, CT, and MRI. Here, we report robot-assisted partial splenectomy for a splenic cyst. Imaging diagnosis of abdominal CT enhancement: the cystic space-occupying of the spleen is considered. We should improve the preoperative examination and exclude operative contraindications. During the operation, there was about 8 cm of the upper pole of the spleen, and the boundary was clear. There was no obvious abnormality in the exploration of the abdominal viscera. The operation was successful. The operative time was 115 minutes, and the blood loss was 20 ml. On the first day after the operation, the patient took a liquid diet. The time of first anal exhaust was on the second day after operation. The patient was discharged at the fourth day. Postoperative pathology revealed epidermoid cyst. The therapy strategy of the splenic cyst is ambiguous. Better understanding of the splenic segmental anatomy and surgical skills has made minimally invasive partial splenectomy a preferred treatment for splenic cysts. In this paper, we report a case of splenic epidermoid cyst managed successfully by robot-assisted partial splenectomy.

## 1. Introduction

Partial splenectomy includes irregular partial splenectomy and regular partial splenectomy. The latter is based on the anatomical basis of sectional blood supply in the spleen. After pretreatment of the two- or three-grade splenic pedicle ligature, the corresponding splenic segment, splenic lobe, or half spleen can be resected according to the ischemic line of the spleen surface. Regular splenectomy is much more difficult than total splenectomy and irregular splenectomy. The main technical points of regular partial splenectomy are as follows: (1) fine dissection and ligation of the second and third splenic pedicle vessels close to the spleen hilum, where each bundle is as small as possible, while observing the spleen blood circulation; (2) moving 0.5-1.0 cm to the healthy side in the relatively nonvascular plane of the spleen and cutting the spleen from a part shallow to a deep part with an ultrasound knife, ligating the blood section (for example, if there are more blood vessels in the splenic section of the spleen, the incision and closure device can be used to cut off the blood vessels); it can be applied to various bleeding measures such as bleeding on the section, wet dressing with warm saline gauze, 8-word suture, adhesives, microwave, and radiofrequency; and (3) peritoneum or splenic capsule transplantation [1].

In recent years, laparoscopic splenectomy has gradually replaced open splenectomy as an important method for the treatment of splenic cysts with the accumulation of laparoscopic surgery experience and the improvement of technology. Compared with the operation time and prognosis, laparoscopic splenectomy has less bleeding and shorter hospitalization time. Compared with laparoscopy, the 3D operation vision and mechanical arm of Da Vinci robotic surgery system overcome the limitation of flexibility in vision and operation instruments, which can greatly enhance the controllability, stability, and accuracy of surgery. Although its application in clinical practice, especially in splenic surgery, is in its infancy, it has been reported that robot-assisted partial splenectomy significantly reduces the anatomical time of the splenic pedicle and overcomes the difficulty of hemostasis and spleen translocation in traditional laparoscopic partial splenectomy and reduces the amount of bleeding [2]. In a research, 24 cases of robot-assisted splenectomy were reported [3]. Among them, 3 cases were partial splenectomy. The median bleeding volume was 75 ml. The average operation time was 199 minutes. The median hospital stay was 5.5 days after the operation. All patients recovered well after the operation [4].

## 2. Case Presentation

**Figure 1:**
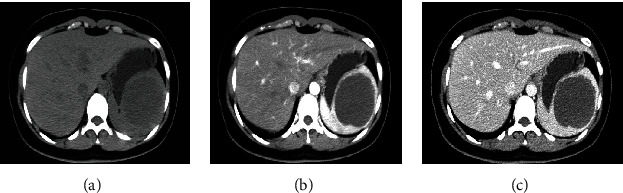
Abdominal CT-enhanced images: (a) shows plain scan; (b) shows arterial phase; and (c) shows venous phase. The upper part of the spleen is about 7-8 cm with slightly low-density shadow.

**Figure 2:**
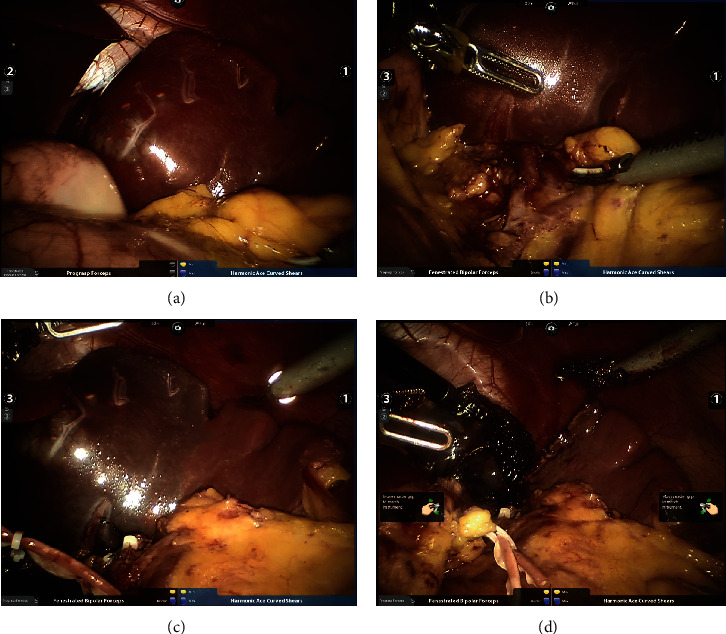
Intraoperative findings: (a) splenic space occupied during operation; (b) free secondary vessels of the splenic pedicle; (c) after disconnection of secondary vessels of the splenic pedicle, ischemic line on the surface of the spleen can be seen; and (d) along the ischemic line, the spleen of an affected side can be removed.

The patient, a 28-year-old woman, was admitted to the Department of General Surgery, Sir Run Run Show Hospital, for half a month of splenic mass found by physical examination. Physical examination showed clear, spiritual, nonyellow skin and sclera, no swelling of superficial lymph nodes, no difference between cardiopulmonary examination, abdominal soft, no obvious mass, normal bowel sounds, no tenderness and rebound pain in the whole abdomen, negative mobile voice, negative Murphy's sign, negativity of renal percussion pain, no edema in both lower extremities, and negative pathological signs. In the imaging diagnosis of abdominal CT enhancement, the cystic space-occupying lesion of the spleen is considered (Figure 1). We should improve the preoperative examination and exclude operative contraindications. During the operation, there was about 8 cm of the upper pole of the spleen, and the boundary was clear.  Figure 2. There was no obvious abnormality in the exploration of the abdominal viscera. The operation was successful. The operative time was 115 minutes, and the blood loss was 20 ml. On the first day after the operation, the patient took a liquid diet. The patient had anal exhaust in the second day and the patient was discharged four days later. Postoperative pathology revealed an epidermoid cyst.

The patient was placed in an incomplete right lateral decubitus position. A pneumoperitoneum of 12 mmHg was established using a Veress needle through a left paraumbilical incision, and an optical port (5- to 12-mm trocar) was introduced afterward. Under visual control, two robotic 8 mm trocars were placed in the left hypochondriac region and the epigastrium. An additional 5- to 12-mm accessory port was placed in the left lumbar region on the middle axillary line for the side assistant surgeon. The surgical cart with the robotic arms was positioned on the patient's left side at a 45° angle to the table's longitudinal axis.

As reported by the European Association for Endoscopic Surgery clinical practice guidelines, in preoperative imaging, splenomegaly was defined with a maximum splenic diameter of more than 15 cm [5]. Postoperative morbidity has been specified as any complication that occurs within 30 days of surgery and has been evaluated as in [6].

**Figure 3:**
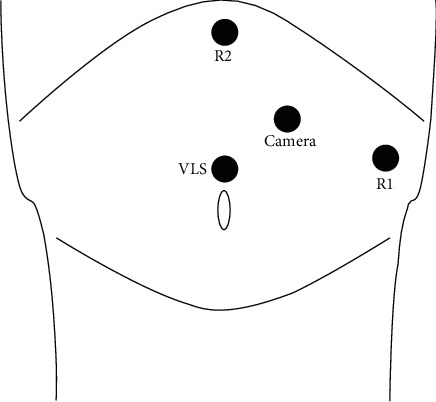
Position of trocars in robotic splenectomy. R1 and R2: robotic arms; VLS: laparoscopic assistance.

Dissection was performed with the robotic EndoWrist® Fenestrated Maryland Bipolar Cautery on the left hand and HarmonicTM Curved Shears on the right hand. The trocar position is presented in Figure 3. The table side assistant elevated and moved the spleen or provided suction if needed. In one case with the large hydatid cyst, it was first inactivated and evacuated under visual control in order to increase the working space in the upper abdomen. After dividing the peritoneal attachments and the splenic ligaments with the HarmonicTM Shears, the omental bursa was opened and the splenic vessels were dissected in the hilum. In the three cases of subtotal splenectomy, the splenic artery and vein were ligated with intracorporeal knot tying using EndoWrist® Needle Holders. After complete mobilization, the splenic parenchyma was transacted using an Endo GIA Roticulator™ blue cartridge stapler introduced by the side assistant surgeon through the accessory port. Vascularization of the splenic remnant was based on the anastomotic branch of the left gastroepiploic pedicle.

The specimen was removed in an Endo Catch™ II 15 mm specimen pouch through the accessory port incision. Hemostasis on the transection surface of the splenic remnant was completed in two cases with a TachoSil® hemostatic sponge.

The drain tube was removed after 1 day. The patient was discharged on postoperative day 4 with no postoperative complication. And the final pathological findings showed epidermoid cyst. We performed a follow-up visit of 1 year, and no recurrence or abnormal platelet count was found.

With the popularization and application of ultrasound, abdominal CT and magnetic resonance imaging in clinical practice, and the awareness of public health examination, the number of splenic cysts diagnosed in recent years has increased significantly. According to the etiology, splenic cysts can be divided into two categories: nonparasitic cysts and parasitic cysts. In nonparasitic cysts, splenic pseudocysts and splenic true cysts can be classified according to the presence or absence of lining epithelium in the cyst wall. Pseudocysts are more common. Most of them are formed after trauma secondary to subcapsular hematoma of the spleen. They are also called secondary splenic cysts. The wall of the cyst is only a fibrous tissue without lining epithelium. The cyst contains blood or serous fluid. The splenic cyst is a rare disease with unknown etiology. The inner wall of the cyst has lining epithelium. The cyst can be unilocular or multilocular. According to pathology, it can be divided into four types: epidermoid cyst, dermoid cyst, cystic lymphangioma, and cystic hemangioma. Ultrasound examination is often the first choice for splenic cysts because of its nonradiation, low cost, and convenient examination. The images are mostly cystic masses with clear borders and dark areas without echoes. After the detection of splenic space-occupying lesions by ultrasonography, CT, MRI, and other imaging examinations can further understand the size, shape, and type of splenic cysts. The number, characteristics of cyst cavity and wall, and adjacent relationship with surrounding organs are of great value.

Surgical treatment is the main method for a splenic cyst. Clinically, nonparasitic splenic cysts less than 2.0 cm in diameter and without any symptoms can be observed regularly. When the diameter of splenic cyst is more than 5.0 cm, the follow-up of the outpatient department increases rapidly or complications such as compression of surrounding organs, rupture, and infection occur; timely surgical treatment is advocated. At present, the main surgical methods depend on the size and location of the cyst, including fenestration and drainage of splenic cyst, total splenectomy, splenectomy with preservation of accessory spleen, hemisplenectomy, and partial splenectomy. As the main immune organ of the human body, the spleen plays the role of regulating immune function and clearing senile blood cells. If total splenectomy is performed, the chance of overwhelming postsplenectomy infection will increase, and complications such as platelet elevation and venous thrombosis will be more likely to occur. Therefore, it is advocated that surgery should preserve the function of the spleen as far as the patient is concerned, such as the presence of a cyst on the upper pole of the spleen or the presence of a cyst at the lower part of the spleen. The diameter of the spleen is less than 50% of the total volume of the spleen. The partial splenectomy or partial splenectomy can be performed according to the specific conditions of the patients.

## 3. Discussion

Splenic cysts are classified into true cyst (primary) and pseudocysts (secondary), on the basis of the presence of an epithelial lining inside the cyst [7]. Splenic true cysts are typically classified as cystic lymphangiomas, cystic hemangiomas, and epidermoid and dermoid cysts. The current consensus is that splenic cysts with a diameter greater than 5 cm or rapid growth rate should be treated surgically to avoid the risk of multiple complications, such as rupture, infection with abscess, and intracavitary bleeding [8].

In recent years, mounting splenic cysts are diagnosed due to the development of modern diagnostic technology. And multiple surgical approaches have been applied in the therapy, especially spleen preserving minimally invasive procedures based on splenic vascular anatomy, which can preserve more than 25% of splenic parenchyma [4]. Partial splenectomy (PS) is now increasingly being promoted, which can reserve the spleen with maximum function to reduce postoperative complications such as thrombocytosis, intra-abdominal abscess, and infections in total splenectomy.

Laparoscopic splenectomy has gradually replace open splenectomy for its safety and minimal invasion. However, a systematic review involving 20 articles elucidated that no statistical differences were observed in many parameters, such as blood loss, time of drainage removal, and incidence of complications between laparoscopy and laparotomy [2].

As a new mini-invasive technique, robotic surgery has shown its superiority in many disease therapies, which is gradually applied in splenology. A robotic surgery system allows for an exquisite dissection due to the high definition and stereoscopic vision and tremor reduction along with perfect maneuverability. These characteristics make it possible to perform complex and advanced surgical procedures. Some surgeons have published their experience with the robot-assisted PS [9, 10].

With regard to the laparoscopic technique, most data focus on laparoscopic decapsulation, while in the literature the feasibility of laparoscopic partial splenectomy for splenic cysts has been well established [10, 11].

A total of 21 cases of the nonparasitic splenic cyst were compared with laparoscopic partial splenectomy. The results showed that intraoperative hemorrhage in the robotic group was significantly less than that in the laparoscopic group. There were no postoperative complications in the robotic group, while 2 cases occurred in the laparoscopic group (left pleural effusion and operative area effusion). In this case, the robot-assisted regular partial splenectomy was performed to prevent the patients from having low comprehensive immunity and more precise excision of the lesion. The procedure was smooth, the field of vision was clear, and the bleeding was less. In practice, it is found that the magnification effect of the robotic surgical system is stronger than that of laparoscopy. It has more advantages for the fine anatomy of the two-degree splenectomy vascular pedicle and can better perform regular partial splenectomy. As a new minimally invasive technique, it has more room for development, though there are still many problems. With problems and controversies, but with the continuous updating of surgical instruments and equipment, the continuous progress of basic and clinical research of spleen, the accumulation of surgical experience, and the improvement of surgical skills, this method of operation will be applied more and more.

While it might be difficult technically to manage partial laparoscopic splenectomy and bleeding from the cut edge of spleen, it can be performed safely with the understanding of the spleen's vascular anatomy. The spleen is divided primarily into two lobes on the basis of vascular distribution. In our case, the demarcation line was formed when the upper lobe vessels were ligated. It is easily accessible without loss of blood for partial splenectomy.

The robot-assisted technique may be the most appropriate for partial splenectomy, as it enables great dissection. The value of partial splenectomy for selected benign diseases in preserving the immune function has been well demonstrated. The comparison of complete and partial laparoscopic splenectomy in children They found that laparoscopic partial splenectomy was as successful as complete laparoscopic splenectomy, although more pain, longer time for oral intake, and longer hospital stay were present in the partial category. The technical challenge associated with partial laparoscopic splenectomy is another factor to consider. The robotic approach is able to overcome the limitations inherent in laparoscopy, especially for hilar dissection and vascular isolation. A partial splenectomy requires effective splenic branch dissection, and in our experience, 3D vision robotic technology and the wrist-like equipment allow good vascular control. In comparison, by applying pledgets, the hemostasis of the cut segment after the parenchymal transection can be performed well. The robot can also be helpful for this challenging task.

## 4. Conclusion

In conclusion, robot-assisted PS is a promising option for the treatment of splenic cysts, which provides an alternative method to the standard laparoscopic approach especially in difficult cases. However, more series and prospective studies are required to further compare the effects of laparoscopic and robotic approaches.
